# Pressure-Tunable Phase Transitions in Atomically Thin Chern Insulator MnBi_2_Te_4_

**DOI:** 10.1021/acs.nanolett.5c05229

**Published:** 2026-01-29

**Authors:** Albin Márffy, Endre Tóvári, Yu-Fei Liu, Anyuan Gao, Tianye Huang, László Oroszlány, Kenji Watanabe, Takashi Taniguchi, Su-Yang Xu, Péter Makk, Szabolcs Csonka

**Affiliations:** † Department of Physics, 61810 Budapest University of Technology and Economics, Müegyetem rkp. 3., H-1111 Budapest, Hungary; ‡ MTA-BME Correlated van der Waals Structures Momentum Research Group, Müegyetem rkp. 3., H-1111 Budapest, Hungary; ¶ Department of Chemistry and Chemical Biology, 1812 Harvard University, Cambridge, Massachusetts 02138, United States; § Department of Physics, Harvard University, Cambridge, Massachusetts 02138, United States; ∥ Department of Physics of Complex Systems, 54616 ELTE Eötvös Loránd University, Pázmány Péter sétány 1/A, H-1117 Budapest, Hungary; ⊥ Wigner Research Centre for Physics, H-1525 Budapest, Hungary; # Research Center for Functional Materials, 52747 National Institute for Materials Science, 1-1 Namiki, Tsukuba 305-0044, Japan; @ MTA-BME Superconducting Nanoelectronics Momentum Research Group, Müegyetem rkp. 3., H-1111 Budapest, Hungary; $ HUN-REN Centre for Energy Research, Konkoly Thege Miklós út 29-33., H-1121 Budapest, Hungary

**Keywords:** topological magnets, pressure-induced phase transition, Chern insulator, topological phase transition

## Abstract

Topological insulators lacking time-reversal symmetry can exhibit the quantum anomalous Hall effect. Odd-layer thick MnBi_2_Te_4_ is a promising platform due to its intrinsic magnetic nature; however, quantization is rarely observed in it. Our magnetoresistance measurements in the antiferromagnetic phase indicate, instead of a quantum anomalous Hall insulator, the presence of a trivial insulator state likely due to disorder, while in a high magnetic field, a Chern insulator state appears. From the magnetic field and temperature dependence, we estimate that the interlayer exchange coupling is enhanced by hydrostatic pressure while the intralayer coupling is weakened. The trivial band gap is also reduced, suggesting the role of disorder is weakened upon compression of the layers.

## Graphical abstract

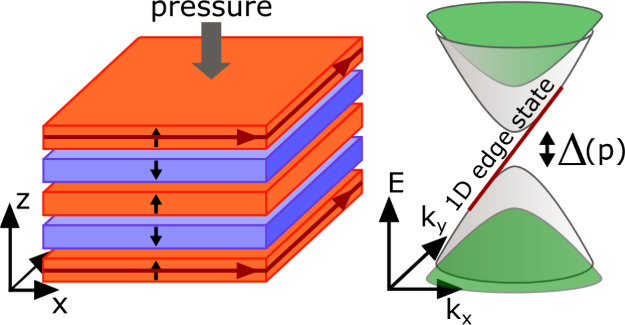

## Introduction

For the quantum anomalous Hall effect (QAHE), a material exhibits a quantized Hall resistance without an external magnetic field, which requires the presence of an intrinsic magnetization that breaks time-reversal symmetry.^1,2^ This effect arises in topological insulators with ferromagnetic order, leading to dissipationless chiral edge states, while the bulk remains insulating. Spatially manipulating such spin-polarized modes is expected to be part of producing novel quantum bits.^3^

MnBi_2_Te_4_ (MBT) is a particularly promising material for the realization of the QAHE because it is an intrinsic magnetic topological insulator,^4–6^ as it naturally combines both topological and magnetic properties without requiring external doping. It is an A-type antiferromagnet (AFM) wherein the magnetization alternates across and is perpendicular to the septuple atomic layers (SLs),^4,7^ as shown in Figure 1a. As van der Waals materials, MBT and related compounds are predicted to be easier to fabricate and control than topological materials doped with magnetic impurities, and they host a wide variety of topological quantum states.^8,9^ Due to the exchange interaction related to the magnetization of the surface layers, the two-dimensional (2D) topological surface states become gapped, as illustrated in Figure 1b. If the Fermi level is tuned into the gap, one-dimensional chiral modes may propagate along the edges of the flake.

**Figure 1:**
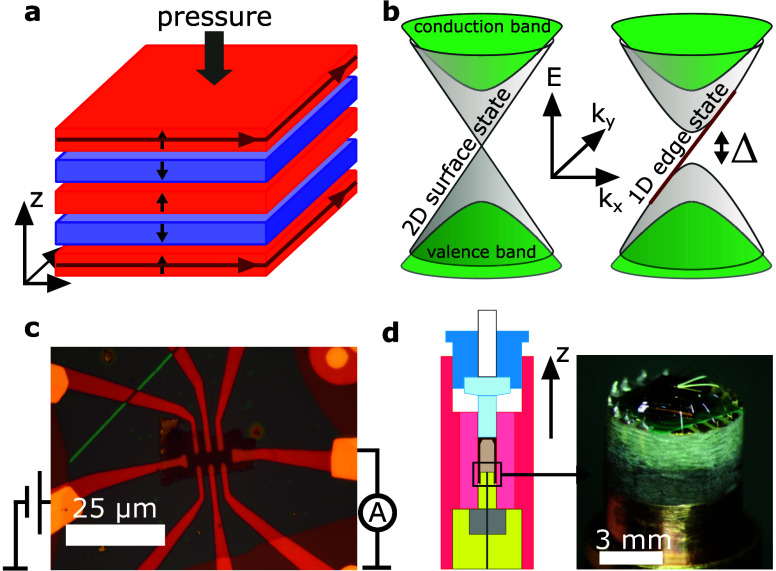
(a) A-Type AFM order for the five SLs. Black arrows represent the SL magnetizations, and dark red arrows the chiral edge states. (b) Illustration of the surface state Dirac cone without (left) and with (right) a topologically nontrivial exchange gap. (c) Optical image of the device. Source and drain contacts are indicated, while the electrical setup of side contacts varied based on the requirements. (d) Schematic of the pressure cell and photo of the PCB carrying the chip.

The characteristics of thin MBT films depend on the number of SLs and the magnetic phase.^10^ In the AFM phase, if the Fermi level is in the gap, odd (even)-SL MBT is expected to exhibit a quantum anomalous Hall (axion) insulator state with unity (zero) Chern number *C*, and the Hall resistance is quantized as |*R*_*xy*_| = *h*/*e* ^2^ ^1,11–13^ (zero^4,6,9,12,14^ ), where *h* is Planck’s constant and *e* is the elementary charge. In a high out-of-plane magnetic field *H*, the layers are fully polarized in a ferromagnetic (FM) phase, and *R*_*xy*_ is similarly quantized in a Chern insulator (CI) state with |*C*| = 1, irrespective of the number of SLs.^9,11,15–21^

However, the QAHE in odd-SL samples is often absent, and quantization may occur only in the field-polarized FM phase. Instead, a wide range of electronic properties has been observed at low field, for example, a topologically trivial insulator state with near-zero *R*_*xy*_.^17,19,20,22,23^ This discrepancy may be related to variations in material quality due to defects or surface degradation,^13,24–28^ potentially affecting the magnetic interactions.^29,30^ Therefore, the goal of this study is to investigate the tunability of the magnetic interactions and the complex phase diagram by reducing the interlayer distance by applying pressure.^31^ We performed magnetoresistance measurements on five-SL MBT samples that lack the QAHE plateau. We studied phase transitions as a function of the magnetic field and the band gaps through thermal activation measurements. We found that the system is highly tunable with pressure *p*. The trivial zero-field band gap is reduced by an increase in *p*, while the CI band gap is weakly affected; moreover, the onset field of the FM phase increases. These observations are consistent with an increase in the interlayer AFM coupling, while the role of disorder appears to be weakened upon compression of the layers.

## Experimental Results

The sample geometry is shown in Figure 1c. The device (detailed in Methods) was fabricated in a glovebox. All MBT samples were protected from air and solvents throughout the whole process. The chip was attached and wire-bonded to a PCB as described in ref 31 and then loaded into a hydrostatic pressure cell as demonstrated in Figure 1d. Measurements were carried out in a liquid helium cryostat equipped with a variable-temperature insert, using a low-frequency lock-in technique. The doped Si/SiO_2_ substrate served as a gate electrode/dielectric. The pressure was applied at room temperature and set for each cool-down. In the test, we show results collected from sample B. Additional data collected on sample A, showing similar trends, can be found in the Supporting Information.

### Electronic Phases

First, we discuss the gate voltage (*V*_g_) and external magnetic field (*H*) dependence of the Hall, longitudinal, and bulk resistances at pressures of approximately 0, 1, and 2 GPa, as shown in Figure 2. *R*_bulk_ was measured by grounding the contacts on both sides of the Hall bar (the three top and three bottom leads in Figure 1c) between the source and drain, effectively eliminating potential edge state contributions to the current.

**Figure 2:**
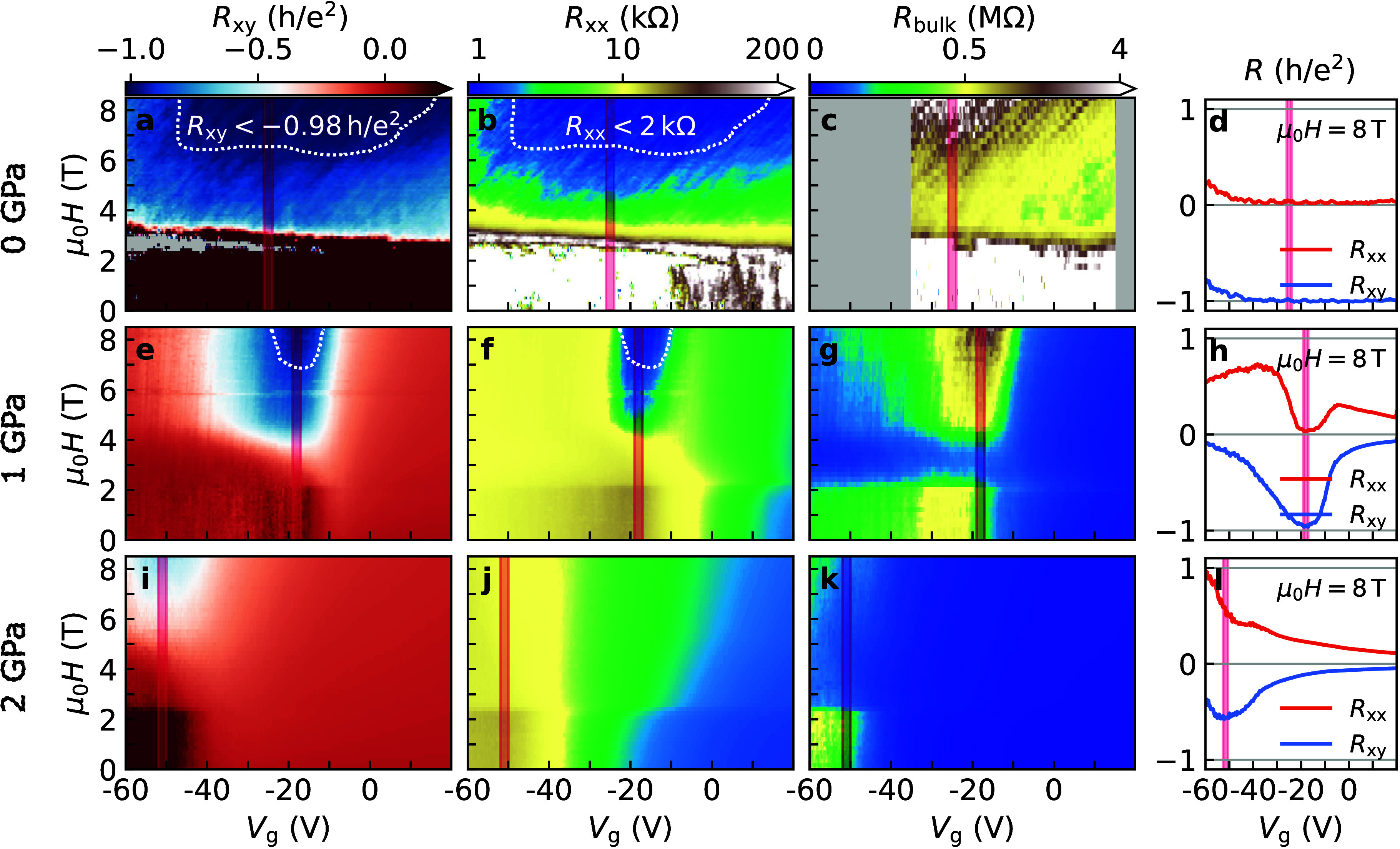
Magnetoresistance at a series of pressures. (a) Map of the Hall (*R*_*xy*_), (b) longitudinal (*R*_*xx*_), and (c) bulk (*R*_bulk_) resistance as a function of gate voltage (*V*_g_) and out-of-plane field μ_0_ *H* at 1.5 K and 0 GPa. (d) Corresponding horizontal cuts at μ_0_ *H* = 8 T of *R*_*xx*_ (red) and *R*_*xy*_ (blue). The same at (e–h) 1 and (i–l) 2 GPa. The CNP estimated at each pressure is marked by red vertical lines. Areas outlined by white dashed lines in panels a and e mark where *R*_*xy*_ ≤ −0.98 *h*/*e* ^2^ and in panels b and f where *R*_*xx*_ ≤ 2 kΩ. For the sake of clarity, the color scale of *R*_*xx*_ and *R*_bulk_ has two linear slopes, with centers at 10 kΩ and 0.5 MΩ, respectively.

Panels b and c of Figure 2 plot *R*_*xx*_ and *R*_bulk_, respectively, as a function of *V*_g_ and *H* at 0 GPa. Their values up to about μ_0_ *H* = 3 T are in the range of hundreds of kiloohms and several megaohms, respectively. This demonstrates that the device is highly resistive in the studied gate range and that there are no edge states, as they would lead to an *R*_*xx*_ below a few kiloohms. Hall resistance *R*_*xy*_, shown in Figure 2a, reaches several *h*/*e* ^2^ in the same region (dark red area), which is caused by mixing with the large *R*_*xx*_ due to the irregular shape of the sample. These observations indicate a topologically trivial insulator state at low fields, similar to the findings of refs 17 and 22. The insulating character is also supported by measurements of *R*_*xx*_ as a function of temperature *T* (see Figure S4).

In contrast, above around μ_0_ *H* = 6 T, the Hall signal becomes quantized, *R*_*xy*_ ≈ −*h*/*e* ^2^, while *R*_*xx*_ decreases close to zero, consistent with a CI state. This region is marked by white dashed lines in panels a and b of Figure 2 and is also demonstrated in panel d as horizontal cuts at 8 T. We estimate the position of the charge neutrality point (CNP) as approximately −25 V and highlight it by vertical red lines in the figure. The presence of a CI state is supported by the increase in *R*_bulk_ at the CNP at high field (Figure 2c), as well as *T* dependence (Figure.S4). In the regime between the trivial and Chern insulator states (∼3–6 T), the reduced *R*_bulk_ suggests a closure or decrease in the bulk band gap, and further discussion of the origin of this phenomena can be found in the Discussion. The quantum Hall effect (QHE) can be ruled out as no fan-like features are visible on the color map, and the sign of *R*_*xy*_ does not change with *V*_g_ at high field.

When the pressure is increased to 1 GPa, features similar to those described above are observed, except in a narrower *V*_g_ range around a CNP of −18 V, as demonstrated in Figure 2e–h. At low field, *R*_*xx*_ and *R*_bulk_ are relatively large, though not as high as at 0 GPa, while *R*_*xy*_ is on the order of 0.1 *h*/*e* ^2^. Including the *T* dependence in Figure S5, these indicate that the system is still a trivial insulator. When the field is increased above ∼2 T, the bulk resistance decreases and then, after an intermediate regime, increases again. This is accompanied by a significant reduction in *R*_*xx*_, while *R*_*xy*_ reaches −*h*/*e* ^2^, heralding the appearance of the nontrivial (CI) state. These areas are again highlighted by white dashed lines. On either side of this *V*_g_ range, the Fermi level is tuned into the valence or conduction bands and the CI quantization disappears (Figure 2h). Compared to the 0 GPa case, the smaller width of insulating or quantized regions along the *V*_g_ axis suggests a decrease in the band gaps or a reduced density of defects.

At 2 GPa (Figure 2i–l), the low-field trivial insulating state is still present with an even lower resistance (see also Figure S6), while the CNP is shifted to −51 V. A quantized *R*_*xy*_ and near-zero *R*_*xx*_ were not observed up to 8.5 T. Nevertheless, there is a peak in *R*_bulk_ in the CNP at high field, and the features in *R*_*xy*_ are similar to the maps at lower pressures. Their tendencies with *V*_g_ and *H* suggest that the FM phase and the corresponding CI state form at magnetic fields that are out of the range of the measurements.

### Magnetic Phases

The longitudinal magnetoresistance and, especially, the anomalous Hall resistance make it possible to determine the magnetic phase transitions. We interpret them in the framework of a linear chain model^11,17,29,30,32^ with the energy function

1 $$E\propto H_{E}\overset{5}{\underset{j=2}{\sum }}M_{j}M_{j-1}-\frac{H_{a}}{2}\overset{5}{\underset{j=1}{\sum }}{M_{j,z}}^{2}-H\overset{5}{\underset{j=1}{\sum }}M_{j,z}$$

detailed in the Supporting Information. Here, *H*_E_ > 0 is the interlayer AFM exchange in units of amperes per meter, *H*_a_ > 0 is the anisotropy that defines the easy (out of plane, *z*) axis, and **M**_*j*_ is dimensionless layer magnetizations of unit length.

We plot *H* sweeps of the symmetrized longitudinal resistance *R*_*xx*(S)_ and antisymmetrized Hall resistance *R*_*xy*(AS)_ close to the CNP in panels a and b of Figure 3. Near zero field, the system is in one of two mirror-symmetric AFM states, illustrated in the bottom of Figure 3d. As the field is increased (black curves in Figure 3), around μ_0_ *H* = 1 T a small decrease in *R*_*xy*_ can be observed at most pressures, highlighted by vertical red lines. This is part of a hysteresis loop between the up and down sweeps (black and red curves, respectively) and is most prominent at 1 GPa. The gate dependence of its magnitude is plotted in panels a and c of Figure 4 and will be discussed further below. It is attributed to the first-order phase transition between the two AFM states and can be observed via the anomalous Hall effect (AHE). This complete flip of the magnetization of all SLs occurs at field *H*_c0_ when the Zeeman energy of the net magnetization becomes large enough compared to AFM exchange *H*_E_ and anisotropy *H*_a_. At 2 GPa, a smaller hysteresis loop can be observed (see also Figure S1a,c), while for 0 GPa, its edge *H*_c0_ could only be determined through conductivity σ_*xy*_ (displayed in Figure S3) due to the contribution of a divergent and noisy *R*_*xx*_ to *R*_*xy*_.

**Figure 3:**
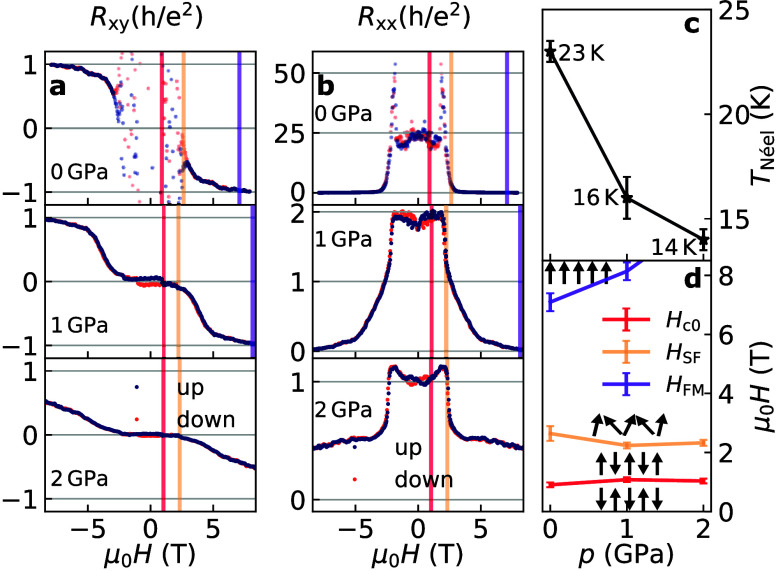
Magnetic transitions. (a) Up (black) and down (red) field sweeps of the antisymmetrized Hall resistance and (b) symmetrized longitudinal resistance (see eqs S1 and S2) close to the CNP for all pressures at 1.5 K. (c) Néel temperature vs pressure based on *R*_*xx*_(*T*) or its derivative (see Figure S2). (d) Estimated transition fields between the magnetic phases vs pressure, as highlighted by colored lines in panels a and b. The orientation of the SL magnetizations between them is illustrated by black arrows.

**Figure 4:**
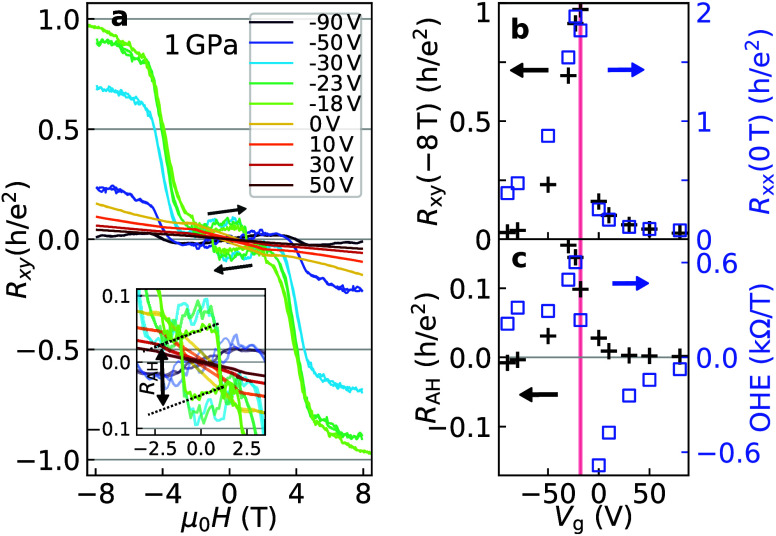
AHE at 1 GPa and 1.5 K. (a) Antisymmetrized *R*_*xy*_ as a function of *H* at a series of gate voltages. The inset shows a close-up of the data, where the dotted lines are linear fits to one of the loops. (b) *R*_*xy*_ at −8 T (black markers) and *R*_*xx*_ at 0 T (blue) as a function of *V*_g_. The CNP is marked by a red line. (c) Size *R*_AH_ of the hysteresis loop (black symbols) and the ordinary Hall coefficient (OHE, in blue) at low field.

Starting from the energetically favorable AFM state above *H*_c0_, further increasing the field produces a suddenly sloping *R*_*xy*_(*H*) and a decrease in *R*_*xx*_(*H*) for all pressures as highlighted by vertical orange lines (*H*_SF_) in panels a and b of Figure 3. The transition can also easily be identified in the nonsymmetrized maps of *R*_bulk_ in Figure 2 near 2–3 T. It is attributed to the spin-flop transition to the canted antiferromagnetic (cAFM) state, which is illustrated by the tilted arrows in Figure 3d. Here all layer magnetizations are partially aligned with the magnetic field, as it dominates the anisotropy, but it still competes with the exchange *H*_E_.

Above 7 T, *R*_*xy*_ and *R*_*xx*_ saturate around −*h*/*e* ^2^ and zero, respectively, as shown in panels a and b of Figure 3 for 0 and 1 GPa. We highlight these fields (*H*_FM_) by vertical purple lines and attribute them to the onset of FM order and the CI state.^11,17^ The transition fields between the phases are plotted in Figure 3d with colors corresponding to the vertical lines in panels a and b. The onset field of the FM phase (*H*_FM_, purple symbols) is much larger than that of the cAFM phase (*H*_SF_, orange) and moves out of range at 2 GPa.

We extracted Néel temperature *T*_N_, which is revealed by a local maximum in *R*_*xx*_(*T*) or can be determined from its derivative (see the Supporting Information and Figure S2), and we show the results in Figure 3c. The estimated value of 0 GPa of 23 K closely matches the values in the literature.^5,17^ *T*_N_ decreases with an increase in pressure, which is consistent with ref 33.

Next, we present the magnitudes of AHE at low and high fields. Figure 4 summarizes the magnetotransport data collected at 1.5 K and 1 GPa (the 2 GPa set is similar (see Figure S1)). In panel a, the antisymmetrized Hall resistance is shown for several gate voltages. As already mentioned in relation to Figure 3, a fully quantized *R*_*xy*_ can be observed at large |*H*| at −18 V, the CNP. Away from the CNP, high-field Hall resistance *R*_*xy*_ (−8 T) decays toward zero as plotted in Figure 4b by black markers. The fact that its sign is independent of *V*_g_ confirms that the plateau is unrelated to the QHE. The position of the peak in *R*_*xx*_(0 T) versus *V*_g_, which is plotted in the same panel in blue symbols, is consistent with the CNP as expected of a low-field insulator state.

In the inset of Figure 4a, a magnified view of the data at low |*H*| is plotted, showing the AHE hysteresis loops. The black dashed lines are linear fits of the −23 V Hall signal. Their vertical distance characterizes the size of the AHE loop, *R*_AH_, as highlighted by the vertical arrow, while their slope gives the ordinary Hall coefficient (OHE). We have estimated these quantities at several gate voltages and plot them in Figure 4c. The low-field OHE (blue markers) crosses zero sharply at the CNP while its magnitude is largest near here. This is consistent with a continuous change from hole to electron transport, marking a difference between the trivial insulator state here and the axion insulator state in even-SL MBT, where it has been suggested that the OHE exhibits a plateau at zero as a function of *V*_g_ near the CNP.^9,27^

*R*_AH_ (black markers in Figure 4c) is largest close to the CNP and decays fast with *V*_g_ on both sides. It changes sign in the hole regime, which is most apparent in the curves at 2 GPa (see the inset of Figure S1a). In other words, at this doping, the low-field AHE signal depends on the net magnetization opposite the high-field case.

### Temperature Dependence

In order to study the nature of the different insulating states as the system is tuned by hydrostatic pressure, we performed temperature-dependent measurements. In Figure 5a, *R*_bulk_ is shown as a function of *V*_g_ and *T* in the FM phase at μ_0_ *H* = 8 T and 1 GPa. It exhibits a maximum value centered near the CNP for all values of *T*. Moreover, it increases with a decrease in temperature, as illustrated by the vertical cut in the CNP in Figure 5b, confirming the presence of a gap in the CI state, Δ_CI_. As for the trivial insulator state, we focus on longitudinal resistance *R*_*xx*_ at 0 T. Figure 5b shows its *T* dependence in the CNP, which enables estimation of trivial insulator band gap Δ_0_. Other measurements for all pressures can be found in the Supporting Information.

**Figure 5:**
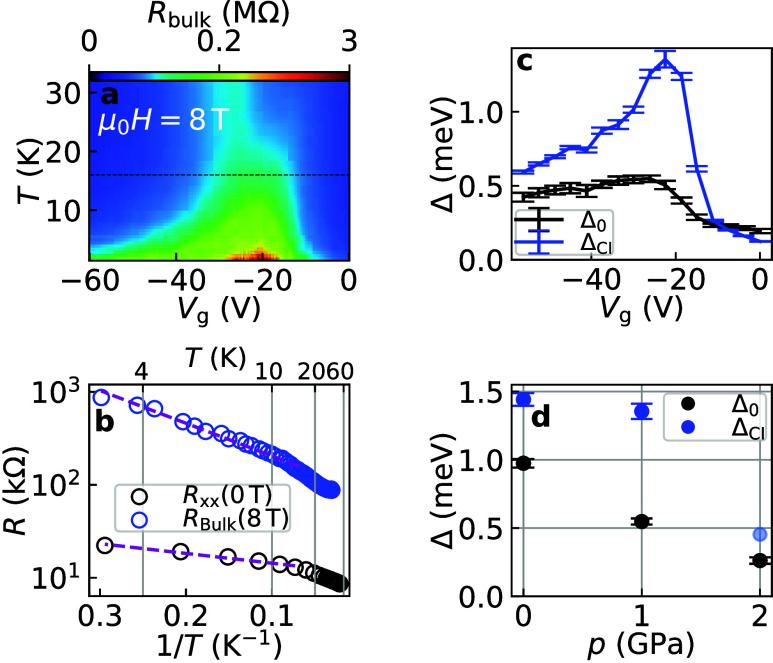
Thermal activation in the trivial and Chern insulator states. (a) Bulk resistance measured at 1 GPa and μ_0_ *H* = 8 T as a function of gate voltage and temperature. For the sake of visibility, the color scale has two linear slopes, with the center at 0.2 MΩ. The black dashed line indicates a *T*_N_ of 16 K. (b) Arrhenius plot of the 0 T longitudinal (black) and 8 T bulk resistances (blue, from panel a) at the CNP. The dashed lines correspond to linear fits of the data below *T*_N_. (c) Thermally activated gap sizes below *T*_N_ as a function of *V*_g_ at 1 GPa. Black and blue markers were obtained from *R*_*xx*_(*T*) and *R*_bulk_(*T*) at 0 and 8 T, respectively, and thus correspond to the trivial (Δ_0_) and CI (Δ_CI_) gap, respectively. (d) Maximal values of Δ_0_ and Δ_CI_ at all pressures.

The gaps were estimated through Arrhenius analysis at all gates, and the results at 1 GPa are plotted in Figure 5c. Both Δ_0_ and Δ_CI_ are maximal close to the CNP. The pressure dependence of their peak values is plotted in Figure 5d. The CI gap is more robust than the trivial gap for all pressures. Δ_CI_ at 2 GPa is likely underestimated, since the FM phase has not yet formed at 8 T. Rather, we expect that it is comparable to the value at 1 GPa. The 0 and 1 GPa points for Δ_CI_ are in the FM phase, and their slowly decreasing trend likely reflects the behavior of the exchange interaction component of the CI gap. In contrast, trivial gap Δ_0_ is significantly suppressed by the pressure.

## Discussion

Altogether, our experimental results show the Chern insulating state at a high field. This state appears in the ferromagnetic phase and forms at larger magnetic fields as the pressure is increased. Our results also show the presence of a trivial insulator state in the AFM phase instead of the QAHE features expected in odd-SL MBT. Ideally, the parallel magnetization of the top and bottom surface layers opens a sizable gap in the topological surface states that, according to theoretical calculations, is on the order of 80 meV.^11,25^ Hence, in simple terms, the characteristic energy scale of QAHE is the exchange energy. However, the gaps extracted are more than 1 order of magnitude smaller. We believe that the absence of the QAHE might be explained in terms of a reduced exchange energy, although the exact mechanism is up for debate.

One possibility that is in good agreement with our measurements is that nontrivial exchange gap Δ_A_ of the AFM state, already strongly weakened by Mn_Bi_ antisite defects as indicated in ref 25, is surpassed by a sufficiently strong disorder potential. This would ultimately lead to a trivial insulator phase,^34^ which has an effective transport gap (Δ_0_). Consequently, intrinsic anomalous Hall conductivity σ_*xy*_ is effectively reduced to near zero (see Figure S3) and strong localization occurs. With an increase in the magnetic field, transitions occur to the cAFM and then to the FM phase. FM exchange gap Δ_CI_ might be larger than Δ_A_ of the AFM phase, since the exchange field is larger if all layers are aligned (discussed further below)^11^ or because an external magnetic field may polarize the Mn antisite defects, effectively increasing the magnetization of each layer.^25^ Hence, Δ_CI_ could dominate the disorder (on the order of Δ_0_), and thus, a magnetic field can induce a quantum phase transition and allow Hall quantization matching our observations. Accordingly, in panels a and b of Figure 3, the change in the slope of *R*_*xy*_ and the decrease in *R*_*xx*_ at the spin-flop (AFM to cAFM) transition at *H*_SF_ indicate the departure from the trivial insulator state and the reappearance of a large intrinsic σ_*xy*_.

The anomalous Hall effect in the AFM state is indeed weak, and interestingly, its magnitude *R*_AH_ changes sign when *V*_g_ is tuned as shown in Figure 4c. This is even more pronounced at 2 GPa, as shown in panels b and c of Figure S1. The sign reversal of the AHE in Cr-doped Bi_2_(Se_*x*_Te_1–*x*_)_3_ samples has been experimentally observed before and is explained in terms of intrinsic AHE in a disordered system.^35^ Another explanation for the sign reversal is an extrinsic origin for AHE.^22^ In any case, the presence of the gate-tunable sign reversal of the AHE supports the possibility of the important role of disorder.

We have analyzed the magnetic transition fields shown in Figure 3d based on the linear chain model (see the Supporting Information). The fact that the spin-flip (cAFM/FM) transition occurs at fields much higher than those of the AFM/AFM/cAFM transitions (*H*_FM_ ≫ *H*_c0_ and *H*_SF_) suggests that easy axis anisotropy field *H*_a_ in eq 1 is significantly weaker than antiferromagnetic interlayer exchange *H*_E_ (see Figure S7b) at all pressures. Moreover, *H*_FM_ increases as a function of pressure while *H*_c0_ and *H*_SF_ remain approximately constant. Based on the model, this is only possible if *H*_E_ increases and *H*_a_ decreases with pressure (see the Supporting Information). This result is consistent with expectations;^23,33^ the former can be attributed to the compression along the *c* axis, and the latter may be the result of the reduced distance of nearest-neighbor Mn atoms due to the compression in the *a–b* plane.^36^ This is in agreement with the evolution of magnetic transition fields under hydrostatic pressure that has been both theoretically analyzed and experimentally observed in related magnetic van der Waals materials.^23,37,38^

As for the Néel temperature shown in Figure 3c, mean field considerations predict that *T*_N_ ∝ *H*_F_ + *H*_E_ in the bulk limit.^39,40^ Here, *H*_F_ is the intralayer ferromagnetic coupling, which would appear in a $-\frac{1}{2}H_{F}\underset{j}{\sum }{M_{j}}^{2}$ term in eq 1 and is assumed to be much stronger than the interlayer coupling (*H*_F_ ≫ *H*_E_). The contribution of anisotropy to *T*_N_ is negligible, in comparison. Therefore, the increase in *H*_E_ with pressure suggests an even greater decrease in *H*_F_. Such an effect has been tied to the intralayer AFM interactions becoming stronger due to smaller Mn–Mn distances in the *a–b* plane^33,36^ and the changing Mn–Te–Mn bond angles, as it effectively decreases the FM coupling within the SL. The effect of disorder on the magnetic coupling strength is considered negligible compared to the influence of pressure-induced structural changes. This interpretation is consistent with our experimental observations. If we assume that the disorder density differs between the two devices, resulting in a small difference in the Néel temperature of about 2 K at ambient pressure, the changes observed under pressure are much larger, clearly indicating that the dominant contribution to the evolution of the magnetic coupling strength arises from the applied pressure rather than from disorder effects.

In light of the above, we can discuss the effect of pressure on the theoretically expected band gaps. The exchange energy of the top or bottom surface of the MBT crystal is ∑_*k*_ *M*_*k*_ *J*_*s*,*k*_, where *J*_*s*,*k*_ is the exchange coupling between the chosen surface and the *k*th layer.^11^ Assuming only intralayer and nearest-neighbor interlayer exchange and using our previous notations, in the FM or AFM phase this sum is proportional to *H*_F_ ± *H*_E_. Neglecting tunneling between the surfaces or disorder, this predicts that the band gap in the FM phase is Δ_CI_ ∝ *H*_F_ + *H*_E_. Its evolution with pressure based on Arrhenius analysis (Figure 5d at 0 and 1 GPa) qualitatively matches the trend of *T*_N_ in Figure 3c. As for the AFM gap, with the above assumptions, it is predicted to be Δ_A_ ∝ *H*_F_ – *H*_E_ and indeed smaller than Δ_CI_, although in the AFM phase disorder is expected to have a more important role, as discussed above, so the above assumption might be less valid. The difference between Δ_A_ and Δ_CI_ may be potentially even wider if the layer magnetizations are affected by field-polarizable defects. Therefore, an intermediate disorder potential may surpass Δ_A_ but not Δ_CI_.

If the disorder is strong enough, then localization occurs in the device. In this respect, we expect that as the lattice constants decrease with pressure on the order of a few percent^33^ and atomic wave functions overlap more and more, localization length ξ increases. Therefore, average activation energy Δ_0_ of hopping transport will decrease.^41–43^ According to Anderson localization, the conductivity increases with localization length: σ_*xx*_ ∝ exp­(−*L*_S_/ξ), where *L*_S_ is the sample size. All of these are in good agreement with our experimental findings (Δ_0_ in Figure 5d and *R*_*xx*_ ∝ σ_*xx*_^–1^ in Figure 2), which suggests that pressure weakens the Anderson insulator state with an increase in localization length. At a sufficiently high pressure, the effect of disorder may become weak enough that it no longer overcomes AFM gap Δ_A_. This would lead to the closure of Δ_0_, the reopening of the nontrivial gap, and the recovery of the QAHE.^34^ However, Δ_A_ also decreases with pressure due to both *H*_F_ and *H*_E_, which may prevent the QAHE state.

While disorder appears to be the dominant limiting factor, subtle surface or interface asymmetry, particularly at the SiO_2_/MBT interface, may also play a small but non-negligible role in influencing the stability of the topological phase.

## Summary

Our study systematically explores the interplay among pressure, magnetism, and topology in a five-SL MnBi_2_Te_4_ film. At low fields in the AFM phase, the near-zero Hall resistance and high longitudinal and bulk resistance indicate a trivial insulator state likely due to disorder. When the FM phase is reached with an increase in the magnetic field, quantization is recovered. From the analysis of magnetic transition fields based on the magnetoresistance, we estimate that interlayer exchange coupling *H*_E_ is enhanced by hydrostatic pressure, while the decreasing Néel temperature suggests that intralayer coupling *H*_F_ is reduced. Although effective trivial band gap Δ_0_ was also reduced, the quantum anomalous Hall effect was not recovered up to 2 GPa, indicating that this pressure is insufficient to fully overcome extrinsic effects such as disorder and interface imperfections.

These findings provide new insights into pressure-driven magnetic and topological transitions in MnBi_2_Te_4_, and they highlight that further advances in material quality and heterostructure design, including improved interface symmetry (e.g., through full hBN encapsulation), may be essential for realizing a robust QAHE in MBT-based systems.

## Supplementary Material



## Notes

Methods, additional experimental data, linear chain model, and transitions in the linear chain model (PDF)
