# Supplementary material for: Pressure-Tunable Phase Transitions in Atomically Thin Chern Insulator MnBi2Te4
Source: Nano Lett. 2026 Jan 29;26(5):1782–8. doi: 10.1021/acs.nanolett.5c05229 (PMC12904095; doi:10.1021/acs.nanolett.5c05229)
Supplement: Supplementary file 1 [file nl5c05229_si_001.pdf]

Supporting Information for Pressure-tunable phase transitions in atomically thin Chern insulator $MnBi_2Te_4$

Albin Márffy,[†,‡] Endre Tóvári,[*,†,‡] Yu-Fei Liu,[¶,§] Anyuan Gao,[¶] Tianye Huang,[¶] László Oroszlány,[‖,⊥] Kenji Watanabe,[#] Takashi Taniguchi,[#] Su-Yang Xu,[¶] Péter Makk,[*,†,‡] and Szabolcs Csonka[†,@]

†*Department of Physics, Budapest University of Technology and Economics, Műegyetem rkp. 3., H-1111 Budapest, Hungary*

‡*MTA-BME Correlated van der Waals Structures Momentum Research Group, Műegyetem rkp. 3., H-1111 Budapest, Hungary*

¶*Department of Chemistry and Chemical Biology, Harvard University, Cambridge, MA 02138, USA*

§*Department of Physics, Harvard University, Cambridge, MA 02138, USA*

‖*Department of Physics of Complex Systems, ELTE Eötvös Loránd University, H-1117 Budapest, Pázmány Péter sétány 1/A, Hungary*

⊥*Wigner Research Centre for Physics, H-1525 Budapest, Hungary*

#*Research Center for Functional Materials, National Institute for Materials Science, 1-1 Namiki, Tsukuba 305-0044, Japan*

@*MTA-BME Superconducting Nanoelectronics Momentum Research Group, Műegyetem rkp. 3., H-1111 Budapest, Hungary*

* E-mail: tovari.endre@ttk.bme.hu; makk.peter@ttk.bme.hu

Methods

MBT crystals were grown by the self-flux method at UCLA.[1] We obtained the thin flakes using standard mechanical exfoliation onto a Si wafer covered with 280-nm-thick SiO_2. We determined the flake thickness by its optical contrast. In order to protect its surface from contamination during the nanolithography process used for fabricating Ohmic contacts, we utilized a shadow mask evaporation technique. The contacted MBT flake was then covered with a thin flake of hexagonal boron nitride (hBN). The whole device fabrication process was done in an argon-filled glovebox, so the air sensitive MBT was protected throughout all steps.

The PCB chip carrier method[2] enables mechanical bonding and a relatively easy and rapid exchange of samples in the pressure cell, compared to directly fixing the chip with epoxy and manually bonding the floating wires. Therefore, the prerequisites for samples (regarding the size and spacing of bonding pads) are less rigorous. Essentially, even samples that were not specifically designed for pressure cell measurements are viable if the lateral size of the chip does not exceed 3 mm.

The (anti)symmetrized longitudinal (Hall) data in Figures 3, 4were calculated following

$$R^{\uparrow\downarrow}_{\mathrm{xx(S)}}(H) = \left[R^{\uparrow}_{\mathrm{xx}}(\pm H) + R^{\downarrow}_{\mathrm{xx}}(\mp H)\right]/2, \tag{S1}$$

$$R^{\uparrow\downarrow}_{\mathrm{xy(AS)}}(H) = \pm\left[R^{\uparrow}_{\mathrm{xy}}(\pm H) - R^{\downarrow}_{\mathrm{xy}}(\mp H)\right]/2, \tag{S2}$$

where $\uparrow$ and $\downarrow$ refer to the direction of the field sweep.

We define the charge neutrality point (CNP) as the gate voltage at which the charge carrier density changes sign. In a system with a trivial band gap, this point typically coincides with a peak in the longitudinal resistance, whereas in a topologically non-trivial system the longitudinal resistance may instead approach zero near the CNP. In our 5-SL MBT samples, the application of an external magnetic field drives a topological phase transition due to the competition between the disorder potential and the exchange gap. Theory predicts that at

sufficiently large disorder potential, the minimal band gap may no longer be located at the Dirac points, implying that different CNPs can appear at low and high magnetic fields. In the present work, we therefore identify only the high-field CNP, using the following criteria: the Hall resistance is closest to quantization; R_{xx} reaches a minimum; R_{bulk} reaches a maximum; and Δ_{CI} is maximized.

Additional experimental data

In this section we present further details of our measurements carried out on sample B (same as in the main article) unless noted differently.

AHE data at 2 GPa: In Fig. S1 the antisymmetrized Hall data at 2 GPa and 1.5 K and its analysis is shown, similarly to Fig. 4 in the main text. Panel (a) shows the anomalous Hall signal at a series of gate voltages. The CI state at high field was not observed, although presumably it appears at higher fields. The hysteresis at low field is still present, and its sign change is especially visible as illustrated in the inset. Fig. S1(b) shows that the Hall resistance at -8 T (black markers) peaks in the vicinity of the CNP, as does the zero-field longitudinal resistance (blue). Panel (c) shows the size of the low-field hysteresis R_{AH} versus the gate voltage (black), and the slope of the linear component of the Hall resistance, the ordinary Hall coefficient OHE (blue). The results are qualitatively similar to those at 1 GPa.

Estimating the Néel temperature: The temperature-dependence of the four-probe resistance at zero field is shown in Fig. S2. Around the Néel temperature T_{N} there are intensified spin fluctuations which add a peak to the expected trend of $R_{\mathrm{xx}}(T)$,[3] which helped determine the Néel temperature plotted in Fig. 3(c). While this peak is apparent at 0 GPa (Fig. S2(a)) in resistances, at higher pressure (b-c) the sloping background makes its identification difficult. Therefore, here we estimated T_N from the center of the $\sim$ shape of the derivatives dR/dT shown by red dashed lines.

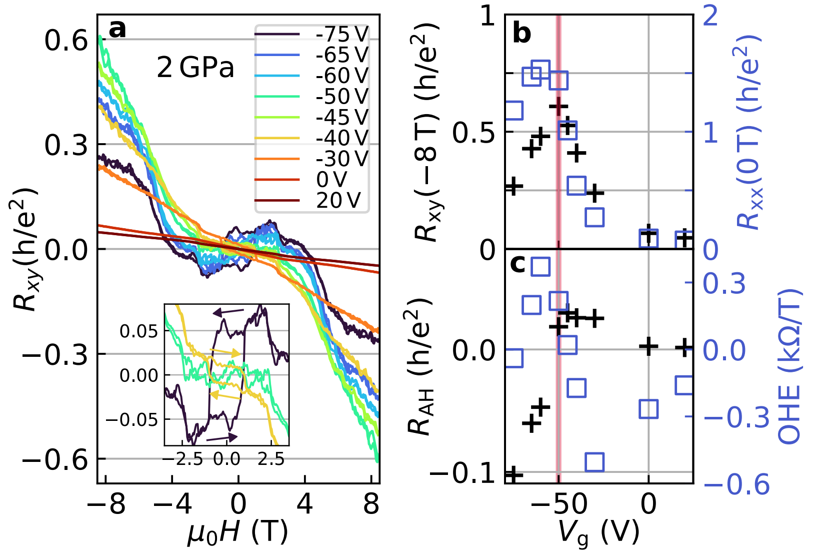

Figure S1: **AHE at 2 GPa and 1.5K. (a)** Antisymmetrized R_{xy} as a function of H at a series of gate voltages. Inset: a zoom of the data. **(b)** R_{xy} at -8 T (black markers) and R_{xx} at 0 T (blue) as a function of V_g. The CNP is marked by a red line. **(c)** The size R_{AH} of the hysteresis loop (black symbols) and the ordinary Hall coefficient (OHE, in blue) at low field.

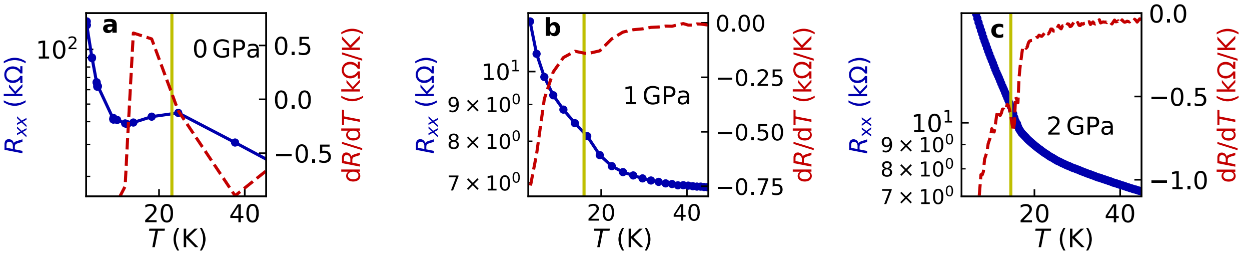

Figure S2: Four-probe resistances and their derivatives (red dashed lines) as a function of temperature for a) 0 GPa, b) 1 GPa and for c) 2 GPa pressure. The antiferromagnetic phase transition manifests as a small resistance peak as a function of T due to spin scattering mechanisms. The value of T_{N} may also be obtained by following the derivative of the resistance, as highlighted by vertical lines.

Extracting the magnetic transition fields: In Fig. S3 we show magnetoconductance curves measured near the CNP at all pressures. While the Hall voltage signal did not diverge at 1 and 2 GPa in the AFM state, enabling the calculation of resistance, it was significantly affected by the mixing of the divergent longitudinal signal at 0 GPa as discussed in the main text. This made the extraction of the two lower magnetic transition fields, H_{c0} for AFM-AFM and H_{SF} for AFM-cAFM (spin flop), difficult. Therefore we estimated them by

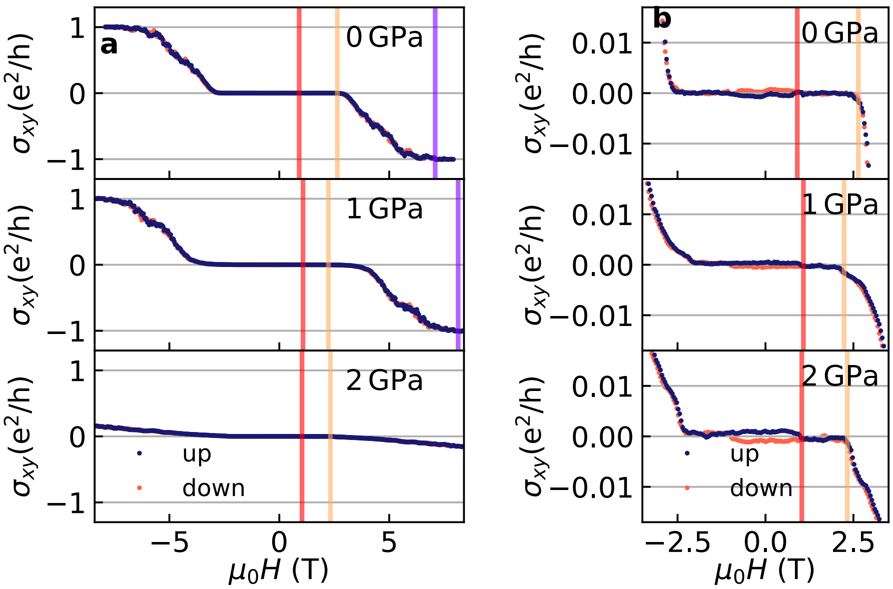

Figure S3: (a) Up (black) and down (red) field-sweeps of the antisymmetrized Hall conductivity close to the CNP at different pressures. (b) Zoom-in to panel (a). Red lines represent the edge of the hysteresis loop (H_{c0}), orange lines the drop in σ_{xy} and the edge of the AFM phase (H_{SF}), and purple lines the onset of FM order (H_{FM}).

calculating the Hall conductivity σ_{xy}, which is plotted in the top panel of Fig. S3 (a) in the full field range, and in the top panel of (b) near zero field. The black and red lines show the up and down sweeps of the field, demonstrating the presence of hysteresis and enabling the extraction of the transition fields. For comparison, σ_{xy} is plotted at 1, 2 GPa as well.

Maps of temperature and gate-dependence: Figures S4, S5, S6 show resistance maps at 0, 1 and 2 GPa, respectively. The longitudinal and Hall resistances as a function of gate voltage and temperature are plotted at 0 T, while R_{bulk} is plotted at 8 T. Below T_N these measurements illustrate the trivial and Chern insulator states without and with magnetic field, respectively. The zero-field R_{xy} at 1 GPa (Fig. S5) clearly shows the disappearance of the AHE in the vicinity of T_N. However, since it carries a component from R_{xx}, this may be hidden, as is the case for 0 and 2 GPa.

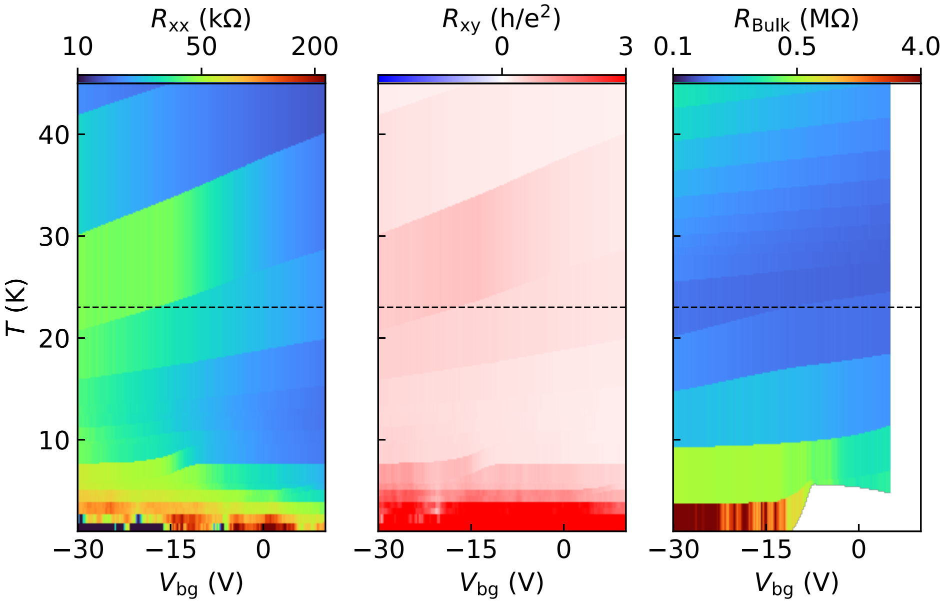

Figure S4: Temperature-dependent resistance measurements as a function of gate voltage at 0 GPa. $R_{xx/xy}$ were measured at 0 T, R_{bulk} at 8 T. The Néel temperature is indicated by a black dashed line.

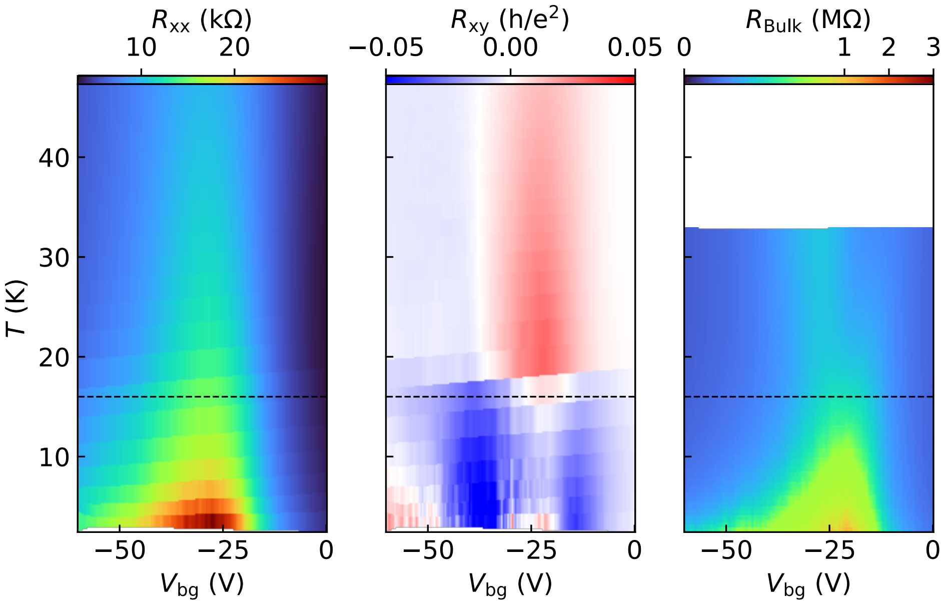

Figure S5: Temperature-dependent resistance measurements as a function of gate voltage at 1 GPa. $R_{xx/xy}$ were measured at 0 T, R_{bulk} at 8 T. The Néel temperature is indicated by a black dashed line.

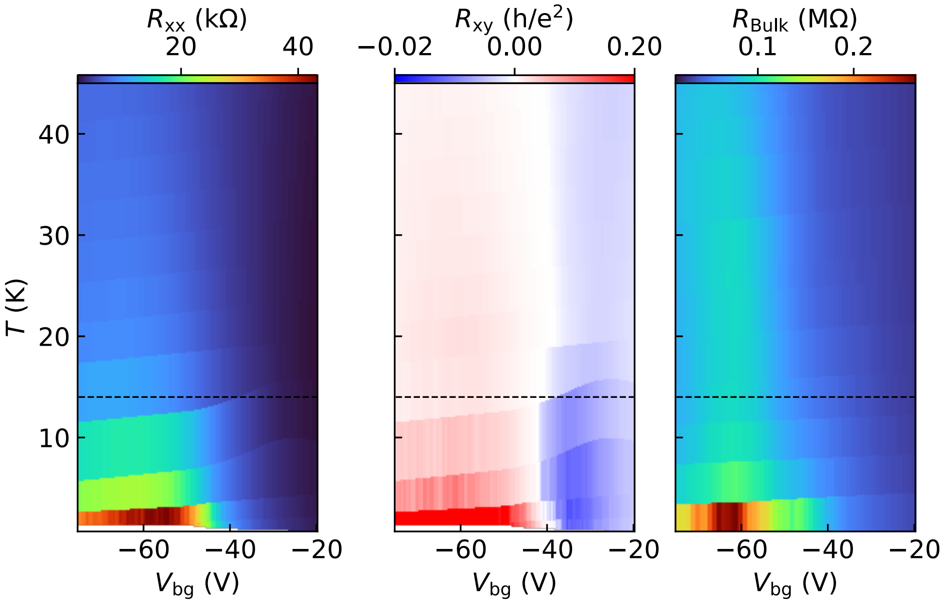

Figure S6: Temperature-dependent resistance measurements as a function of gate voltage at 2 GPa. $R_{xx/xy}$ were measured at 0 T, R_{bulk} at 8 T. The Néel temperature is indicated by a black dashed line.

Additional data of device A: Figure S7, S8, S9, S10, S11 shows data collected on an additional device, device A. The general quality of device A is worse than that of device B since at zero pressure even in high magnetic field the Hall signal diverges and the longitudinal resistance is in the order of 10 MΩ (beyond the limitation of lock-in technique) instead of a small value characteristic to edge states. At higher pressures (1 and 2 GPa) we observed a substantial drop both in R_{xx} and in R_{xy} as it can be seen on figure S8 and S9 compared to the 0 GPa case.

On fig. S10 R_{xx}, the two terminal resistance R_{2T} and their derivatives are shown as the function of temperature for various pressures. Following these $R(T)$ curves the Néel temperature can be obtained which we show on fig. S11(c) as the function of pressure which is 11 K, 13 K, 21 K for 0 ,1 and 2 GPa respectively. Similarly to device B the Neél temperature decreases with pressure.

On fig. S11(a) and (b) we show the symmetrized device magnetoresistances close to the CNP for different pressures. Generally a strong decrease in resistance with pressure can be observed. The Hall resistance at zero pressure is so large that the data is very noisy at that pressure, whereas at 1 GPa the Hall resistance at $\mu_0 H \approx 8\,\mathrm{T}$ is close to quantization and a small Anomalous Hall effect loop can be observed at small magnetic field. At $p = 2\,\mathrm{GPa}$ the longitudinal resistance further decreases while both the low and high field Hall signal becomes smaller, qualitatively agreeing with those results obtained from the measurements of device B (main article). Fig. S11(d) shows the obtained critical magnetic fields as the function of pressure. On this figure a similar trend of the parameters can be observed compared to the case of device B, although H_{FM} can not be determined because in the investigated magnetic field range the device shows no quantization.

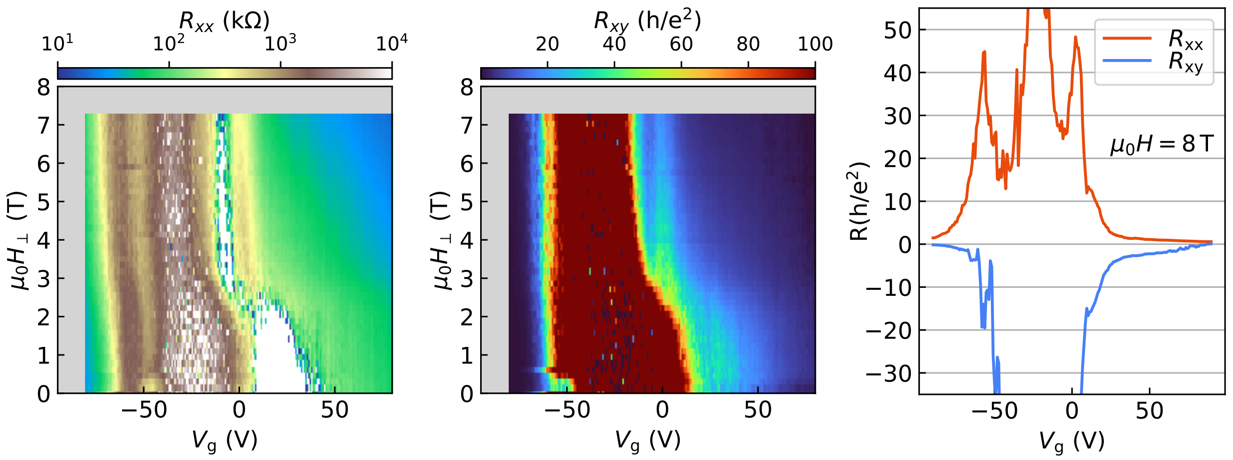

Figure S7: **Magnetoresistance at 0 GPa pressure** (a) Map of the longitudinal (R_{xx}), (b) the Hall (R_{xx}) resistance as a function of gate voltage (V_g) and out-of-plane field $\mu_0 H$ at 1.5 K and 0 GPa. (c) Corresponding horizontal cuts at $\mu_0 H = 8\,\mathrm{T}$ of R_{xx} (red) and R_{xy} (blue)

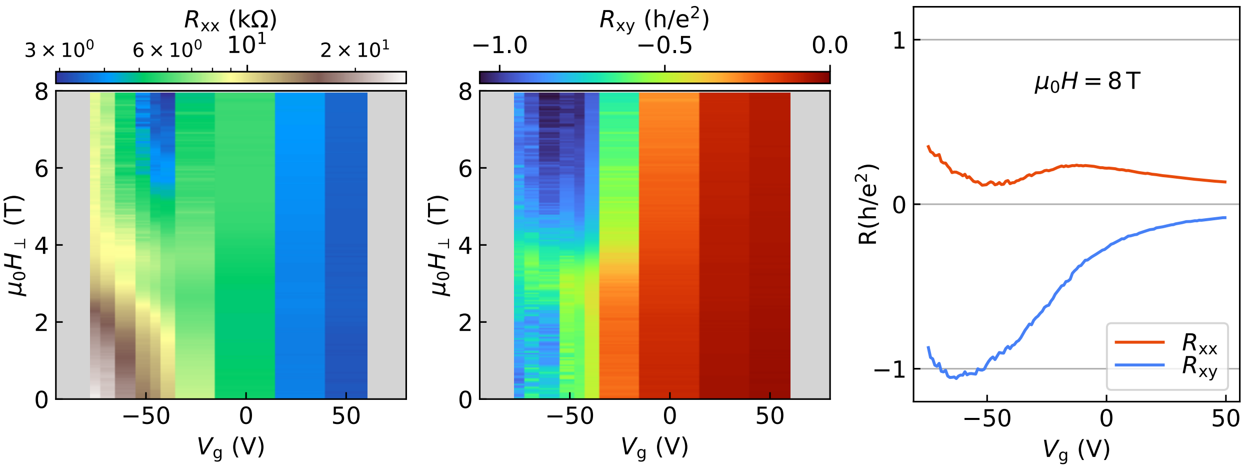

Figure S8: **Magnetoresistance at 1 GPa pressure** (a) Map of the longitudinal (R_{xx}), (b) the Hall (R_{xx}) resistance as a function of gate voltage (V_g) and out-of-plane field $\mu_0 H$ at 1.5 K and 0 1Pa. (c) Corresponding horizontal cuts at $\mu_0 H = 8\,\mathrm{T}$ of R_{xx} (red) and R_{xy} (blue)

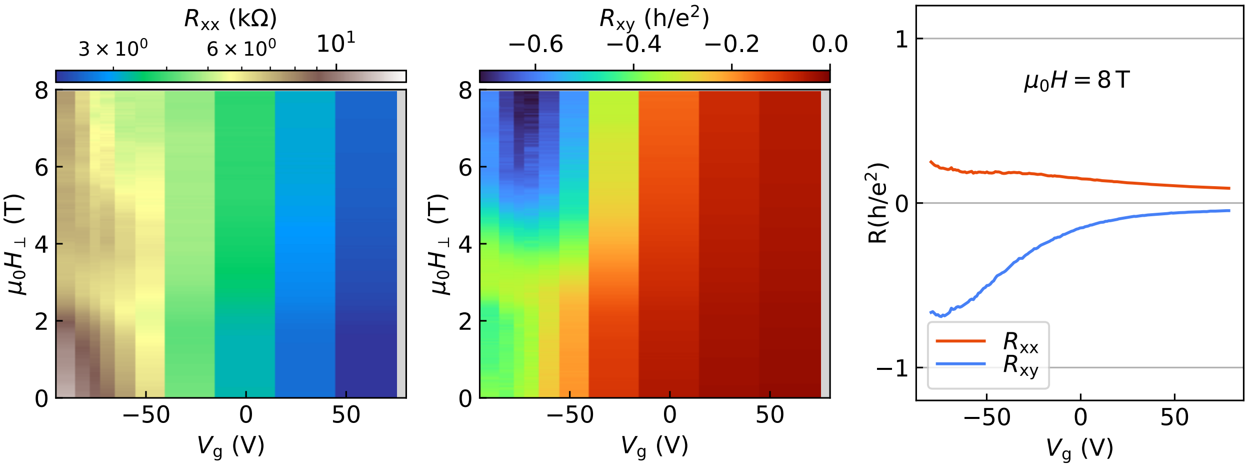

Figure S9: **Magnetoresistance at 2 GPa pressure** (a) Map of the longitudinal (R_{xx}), (b) the Hall (R_{xx}) resistance as a function of gate voltage (V_g) and out-of-plane field $\mu_0 H$ at 1.5 K and 1 GPa. (c) Corresponding horizontal cuts at $\mu_0 H = 8\,$T of R_{xx} (red) and R_{xy} (blue)

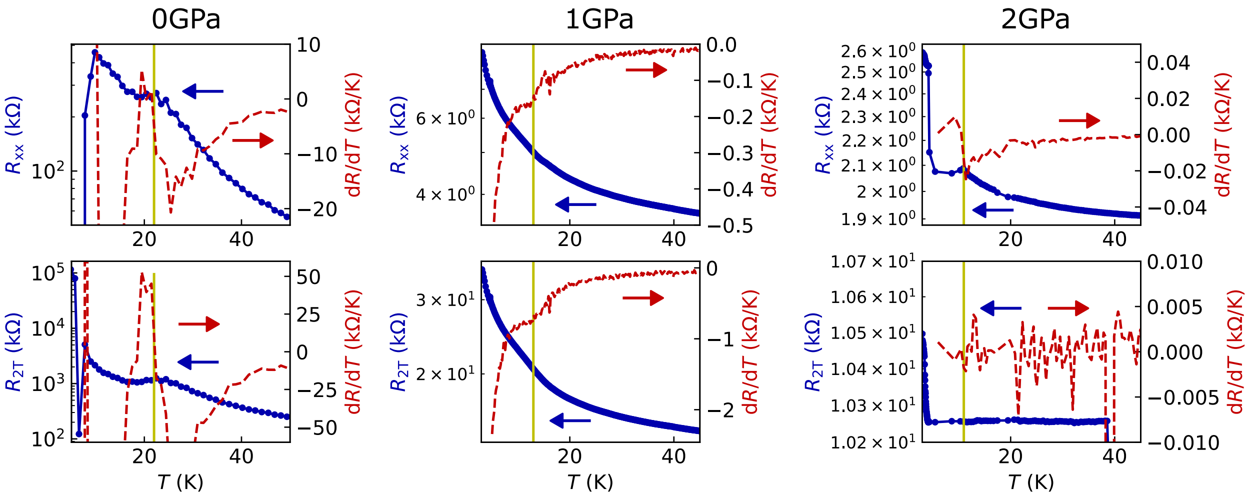

Figure S10: Four-probe and two-terminal resistances of device A and their derivatives (red dashed lines) as a function of temperature for 0 GPa, 1 GPa and for 2 GPa pressure. The antiferromagnetic phase transition manifests as a small resistance peak as a function of T due to spin scattering mechanisms. The value of T_N may also be obtained by following the derivative of the resistance, as highlighted by vertical lines.

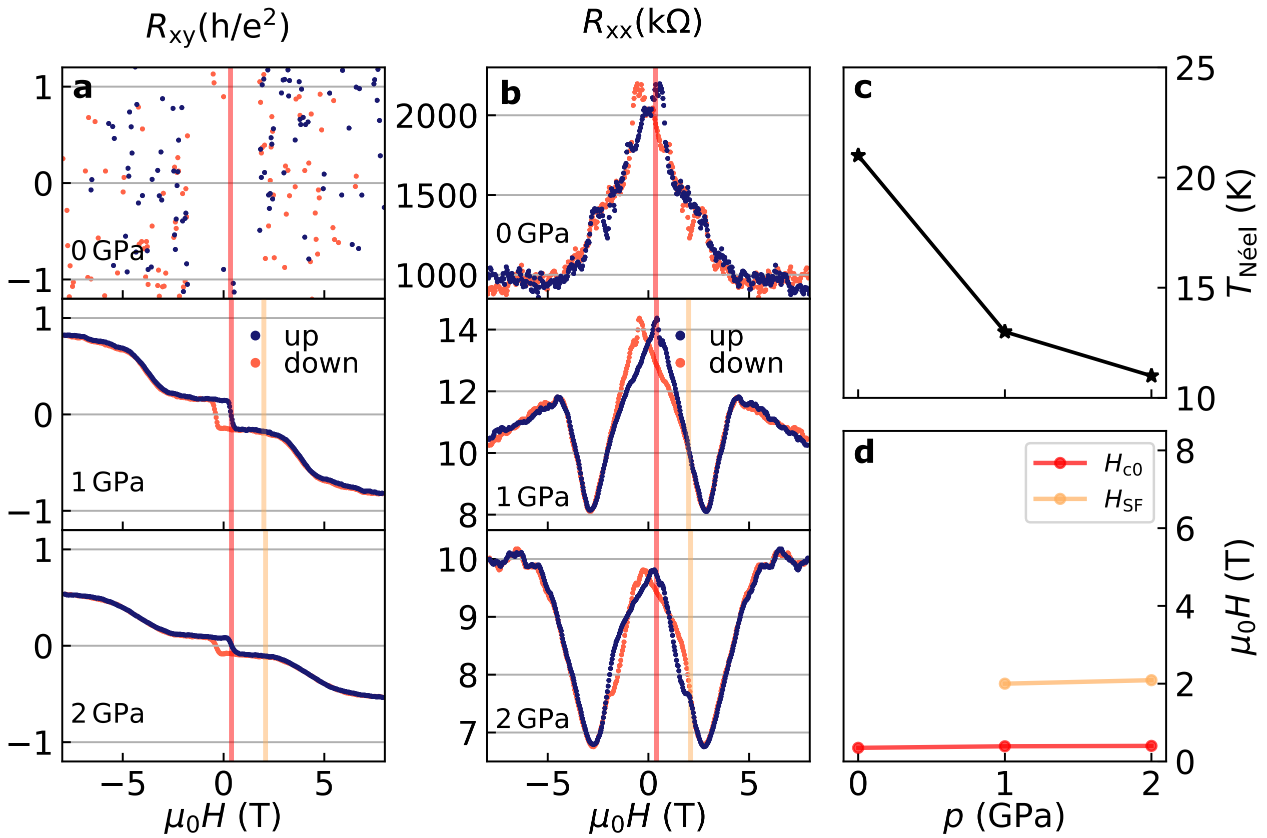

Figure S11: **Magnetic transitions in device A** (a) Up (black) and down (red) field-sweeps of the antisymmetrized Hall resistance and (b) the symmetrized longitudinal resistance (see Eqs. 2, 3) close to the CNP for all pressures at 1.5 K. (c) Néel temperature vs pressure based on $R_{xx}(T)$ or its derivative (see Fig. S10). (d) The estimated transition fields between the magnetic phases vs pressure, as highlighted by colored lines in (a,b).

The linear chain model

An effective classical model similar to the Stoner-Wohlfarth model can be used to describe the magnetic phase transitions in an A-type AFM like MBT. In this section we discuss the model and the magnetic phases and transition fields predicted by it. In the next section we will use it to estimate the interlayer AFM coupling H_E and the anisotropy H_a and demonstrate their pressure-dependence.

In MBT the intralayer FM coupling is relatively strong, which enables describing each layer with a macrospin magnetization, saturated in the low-temperature limit. We assume an easy-axis anisotropy along the z axis (out of plane for MBT) and set a magnetic field parallel to it. Therefore we need only consider the orientation of each layer, and the energy function can be simplified to the following form:[4–8]

$$f = H_E \sum_{j=2}^{N} \cos(\varphi_j - \varphi_{j-1}) - \frac{H_a}{2} \sum_{j=1}^{N} \cos^2 \varphi_j - H \sum_{j=1}^{N} \cos \varphi_j. \tag{S3}$$

Here $\varphi_j \in [0, 2\pi[$ is the magnetization orientation of the jth layer relative to z, N is the layer number, $H_E > 0$ is the interlayer AFM exchange, $H_a > 0$ is the anisotropy energy selecting z ($\varphi = 0$ or π) as the easy axis, and the final term is the Zeeman term.

Analytical solutions

To determine the borders of the magnetic phases, we need to consider the gradient of f, as well as the Hessian matrix with elements $A_{jk} = \partial^2_{\varphi_j,\varphi_k} f$. A is a tridiagonal matrix where the super, sub, and main diagonals are, respectively,

$$A_{j,j+1} = H_E (1 - \delta_{jN}) \cos(\varphi_{j+1} - \varphi_j) = b_j, \tag{S4}$$

$$A_{j,j-1} = H_E (1 - \delta_{j1}) \cos(\varphi_j - \varphi_{j-1}) = d_j, \tag{S5}$$

$$A_{j,j} = -(b_j + d_j) + H_a \cos 2\varphi_j + H \cos \varphi_j = a_j \tag{S6}$$

where δ_{jk} is the Kronecker-delta.

$$A = \begin{pmatrix} a_1 & b_1 & & & \\ d_1 & a_2 & b_2 & & \\ & d_2 & a_3 & \ddots & \\ & & \ddots & \ddots & b_{N-1} \\ & & & d_{N-1} & a_N \end{pmatrix}. \tag{S7}$$

We will use the eigenvalues of this matrix in the various magnetic phases that satisfy $\boldsymbol{\nabla} f = \mathbf{0}$ to determine the field regimes where they provide local minima in the energy.

We shall denote the phases by the same acronyms as before, but specify their net magnetization by a subscript. For instance, FM_{+N} will mean the fully polarized state where all layer magnetizations are aligned with a positive field H (↑↑↑↑↑ for $N = 5$). The net dimensionless magnetization, defined as

$$M = \sum_{j=1}^{N} \cos \varphi_j, \tag{S8}$$

is $M = +N$ in this phase (the saturation magnetization of a SL is taken as 1).

AFM-like phases:

For an even number of layers we label the antiferromagnetic state near $H = 0$ as AFM_0, since $M = 0$ (for $N = 4$, it is ↓↑↓↑ or its reverse). For odd N, the states $\text{AFM}_{\pm 1}$ compete at low $|H|$: AFM_{+1} is where $(N + (-)1)/2$ layer magnetizations are (anti)parallel with H, and AFM_{-1} is the reverse. For $N = 5$ they are ↑↓↑↓↑ and ↓↑↓↑↓. We will also use the notation AFM for AFM-like states where SL magnetizations are along $\pm z$ but not fully polarized ($|M|$ is an integer and $< N$).

cAFM and FM:

For weak anisotropy the canted AFM (cAFM) phase is possible. In this state the magnetic

moments are not parallel with the easy axis or the applied field, and due to the AFM coupling, their orientation alternates from layer to layer, like ↖↗↖↗↖ for $H > 0$ and $N = 5$. If we start increasing H from the cAFM$_+$ phase (+ indicates $M > 0$), the system eventually reaches the fully polarized FM$_{+N}$ state (↑↑↑↑↑). The transition to FM$_{+N}$ is second-order, the cAFM-FM transition or its reverse does not produce a hysteresis. This saturation field $H_{\rm FM}$ is the edge of stability of the cAFM$_+$ state when increasing H. It is also the edge of stability of the FM$_{+N}$ state when decreasing H. In general, the edge of stability means that here the corresponding local minimum in f disappears while changing H and the system must find another. In this example (cAFM$_+$ to FM$_{+N}$ or vice versa), the local minimum does not disappear and there is a seamless (hence second-order) transition. A magnetization curve simulated for $N = 5$ is illustrated in Fig. S12(f) by a black line (simulations are discussed further below), the saturation at $H_{\rm FM}$ is indicated by the purple line.

$H_{\rm FM}$ can be determined by calculating the (positive) eigenvalues of A in the FM$_{+N}$ state, and find where the smallest of them reaches zero when decreasing H. Since $\varphi_j = 0$, the matrix elements are $b_j = d_j = d = H_E$, $a_{j=2:N-1} = a = -2H_E + H_a + H$ while $a_{j=1,N} = a+d = -H_E+H_a+H$ due to only a single neighboring layer. Using trial eigenvectors with k^{th} components $u_k = e^{\gamma k} + c \cdot e^{-\gamma k}$, the solutions are of the form $\gamma_m = (m-1)\,\pi i/N$ where i is the imaginary unit, and the eigenvalues are $\lambda_m = H_a + H - 2H_E \left(1 - \cos\frac{(m-1)\pi}{N}\right)$. The smallest is λ_N, therefore enforcing $\lambda_N = 0$ gives the edge of stability of the FM$_{+N}$ state, which is also the border of the cAFM$_+$ phase. As stated above, the field where it occurs is the saturation field

$$H_{\rm FM} = H_{\rm FM0} - H_a, \text{where} \tag{S9}$$

$$H_{\rm FM0} = 4H_E \cos^2\frac{\pi}{2N}. \tag{S10}$$

Here $H_{\rm FM0}$ is the saturation field at $H_a = 0$, which is consistent with Ref. 9. The saturation field for the opposite, FM$_{-N}$ state is $-H_{\rm FM}$: the edge of stability for this state when increas-

ing H, and the edge of stability for the cAFM$_-$ state when decreasing H. This is visible in Fig. S12(f) in the symmetry of the saturated regions of the simulated magnetization (black line). These borders are plotted as diagonal purple lines in Fig. S12(a) for not too high anisotropy.

As for stronger anisotropy, above a certain H_a the cAFM phase is no longer a *global* energy minimum for any H. Here we determine this critical value of H_a. In this regime the global minimum of the AFM$_0$ or the AFM$_{+1}$ state (depending on the parity of N) becomes equienergetic with that of the FM$_{+N}$ state at a field we label as H_{AFM}, which is H_E for $N = 2$ layers and $2H_E$ for $N > 2$. The critical H_a^c above which the cAFM state cannot be a global minimum is determined by the equation $H_{\mathrm{FM}}(H_a^c) = H_{\mathrm{AFM}}$, producing

$$H_a^c = \begin{cases} H_E & \text{if } N = 2, \\ 2H_E\left(2\cos^2\frac{\pi}{2N} - 1\right) & \text{if } N > 2. \end{cases} \tag{S11}$$

Above H_a^c the border of the FM phase being a global minimum is H_{AFM}, independent of H_a, which is indicated by the purple lines turning vertical in Fig. S12(a).

Numerical calculations

Looking for the global energy minimum:

We have numerically simulated the possible magnetic configurations. We performed a global minimum search in the phase space $\{\varphi_j\}$ for several values of H and H_a by using a brute force approach combined with the gradient method, optimized to the problem by exploiting the symmetries of Eq. S3. The results are shown in Fig. S12(a) by plotting the magnetization M in a colormap as a function of H/H_{FM0} and H_a/H_a^c. The numerical approaches match well the analytical calculations: the cAFM state is indeed a global minimum between the purple lines up to $H_a = H_a^c$, the FM states occur outside these lines, while at low H the AFM states are the most stable. It also gives the AFM-cAFM boundaries

according to global minimum considerations, which is not possible analytically. An example of the solutions along a horizontal cut at $H_a/H_a^c = 0.5$ are shown in Fig. S12(b,c). The $\mathrm{AFM}_{\pm 1}$ states with $M = \pm 1$ are easily recognizable. Panel (b) demonstrates that in the cAFM phase the angles φ_j of next nearest neighbor layers (1-3, 2-4 or 3-5), while similar, are not equal. Therefore a two-sublattice model would be inadequate to describe a system with such a low number of layers. We note that for an even number of layers and $N > 2$, the global minimum map is similar but has three low-H regions instead of one: for states $\mathrm{AFM}_{-2,0,+2}$ from left to right, except for very low H_a where only a narrow AFM_0 region may exist (not shown).

Looking for local energy minima:

In order to get a better picture about possible hystereses, first we analytically calculated the stability boundaries (using the eigenvalues of the Hessian A) of the AFM and the AFM-like states which possess $M = \pm 1, \pm 3$. These were determined by finding $\nabla_\varphi f = 0$ as well as A being a positive definite matrix. The results are plotted as colored lines in Fig. S12(d): orange and red for the $\mathrm{AFM}_{\pm 1}$ states, and magenta for the AFM-like states $\mathrm{AFM}_{\pm 3}$ which have the configurations ↑↑↓↑↑ and ↓↓↑↓↓. Within these regions (above the lines) the states are local minima in the energy landscape. We note that the configurations ↓↑↑↑↑ and ↑↓↑↑↑ (and similar ones with reversed spins or reversed order) also produce $M = 3$ (or -3) but are contained within the regions outlined in magenta. The border between the cAFM and FM states is $\pm H_{\mathrm{FM}}$, plotted by purple lines. The FM states are stable for H outside these lines, while cAFM states can be stable between them, although not everywhere.

Besides the above analytical calculations, we performed numerical simulations at a series of fields H at different H_a to find the local energy minima in $\{\varphi_j\}$ space. First we selected the value of H_a and the H sweep direction, then set the starting point of H below $-H_{\mathrm{FM}}$ or above $+H_{\mathrm{FM}}$, depending on the direction. The starting state $\{\varphi_j\}$ was the corresponding FM state, for example FM_{-N} for an upsweep. After each incremental change in H, the

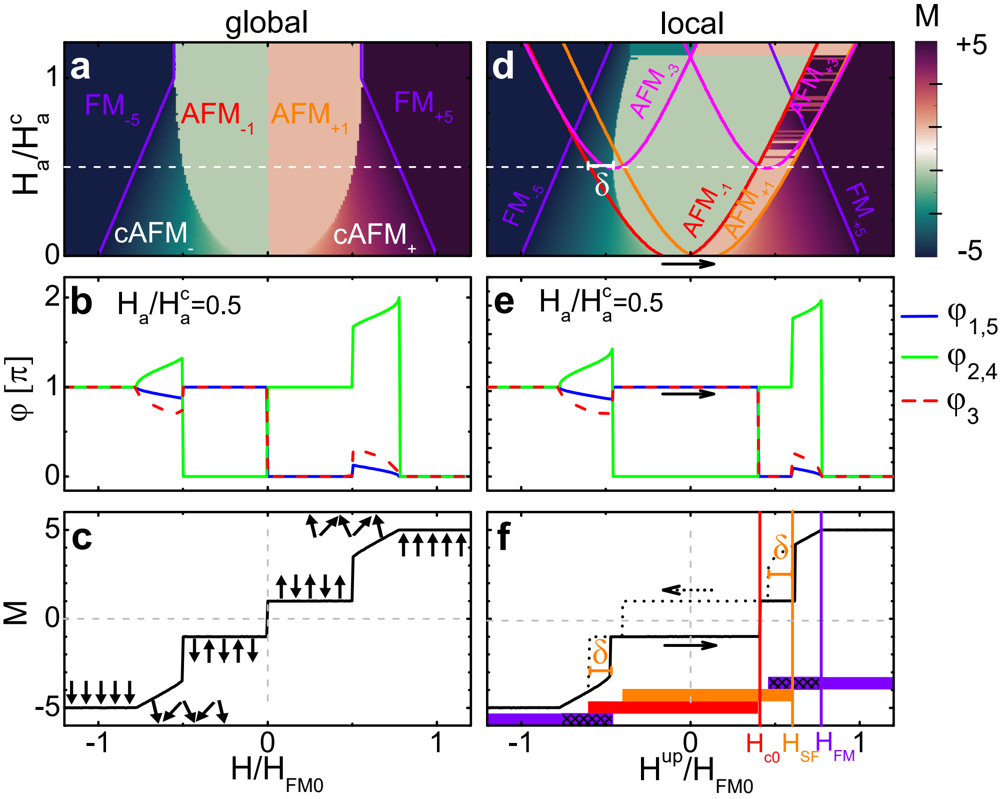

Figure S12: Magnetic simulations for $N = 5$. (a) Numerical global energy minimum search result showing a colormap of M/M_{sat} as a function of the dimensionless field H/H_{FM0} and anisotropy H_a/H_a^c. The purple lines are the borders of the FM phases. (b) The angles φ_j of individual layer magnetizations, and (c) the net easy axis magnetization M/M_{sat} from a global minimum search, along the white dashed line ($H_a/H_a^c = 0.5$) in (a). (d) Numerical local minimum search result while increasing H (upsweep). Lines show the analytical stability borders of various phases based on the Hessian A. (e) The angles and (f) the net magnetization during an upsweep at $H_a/H_a^c = 0.5$. The latter also shows the corresponding downsweep as a dotted line. The colored rectangles highlight the stability regions of the states: purple for FM, cross-hatched purple for cAFM, orange and red for $\mathrm{AFM}_{\pm 1}$.

gradient and the Hessian were checked to see if the latest energy minimum became unstable. If it did, a random walk was performed until the gradient method could converge to another local minimum. The results for an upsweep of H are plotted in Fig. S12(d) by a colormap of M, and the specific solution at $H_a/H_a^c = 0.5$ is shown in panels (e,f). In Fig. S12(f) the downsweep is also plotted, demonstrating hysteresis loops at the AFM-AFM and AFM-cAFM transitions, the latter's size denoted by δ in panels (d,f).

Let us follow M during the upsweep along Fig. S12(d) and (f): the right edge of the

FM_{-5} state in the simulation matches the analytical $-H_{\rm FM}$ (purple line in (d)) without a hysteresis loop since the FM-cAFM transition is second-order. Next we arrive at the right edge of the $cAFM_{-}$ state at an analytically unknown field and switch to the only possible minimum, AFM_{-1}. Its right edge is correctly predicted from the Hessian (red lines in (d,f) denoted by $H_{\rm c0}$) and the system switches to the AFM_{+1} state. The right edge of this is also correctly predicted (orange lines, $H_{\rm SF}$). The following $cAFM_{+}$ state seamlessly turns into FM_{+5} at $+H_{\rm FM}$ as expected (purple again). The stability regions of the phases based on the calculations are also highlighted in the bottom of Fig. S12(f) by colored rectangles.

At higher H_a, when a state destabilizes upon increasing H, multiple local minima may be possible. As a result the $AFM_{\pm 3}$ states can also be randomly observed in the numerical simulation (Fig. S12(d)) within the predicted regions, with their right edges matching the calculated curves (magenta lines). The downsweep map may be produced without running a simulation: it is given by $M^{\rm down}(H) = -M^{\rm up}(-H)$, which is illustrated by an actual calculated downsweep curve in Fig. S12(f) (dotted line). The exception is when there are multiple available local minima which introduce randomness into the picture. Based on Fig. S12(d) this may occur above $H_a/H_a^c > 0.5$.

Transitions in the linear chain model

Here we discuss the experimentally observed phase transitions in light of the linear chain model. When plotting the transition fields, we always follow the same colors as in Fig. 3 in the main text, see for example Fig. S12(f).

Regarding the $AFM_{\pm 1}$ phases, a hysteresis and a finite switching field $H_{\rm c0}$ between them are expected. They are, indeed, observed in experiment. However, at the start of the cAFM phase (spin-flop field $H_{\rm SF}$, orange lines in Fig. 3 and Fig. S12(f)) we expect a sudden jump in $R_{\rm xy}$ due to the abrupt change in the net magnetization, but only observe a change in its slope. This discrepancy may be due to layer-dependent magnetic parameters (potentially

from defects), the presence of domains,[7] or that here $R_{\rm xy}$ is likely dominated by an intrinsic AHE which is not exactly proportional to M. Moreover, based on Fig. S12(f), we expect different $H_{\rm SF}$ values between up- and downsweeps of H, i.e. a hysteresis between the AFM and cAFM phases. In contrast, the magnetoresistance curves lack a hysteresis, but we should notice in Fig. S12(d) that the expected width δ of this hysteresis loop becomes negligible below $H_a/H_a^c \approx 0.25$. As we shall see, the parameters truly fall in this range.

Qualitative evaluation of H_E, H_a versus pressure:

First, we explain the qualitative assessment in the main text about the change of magnetic parameters H_E, H_a with increasing pressure. Fig. S13(a) shows in solid symbols that the experimental $H_{\rm FM}$ increases with pressure, while $H_{\rm c0}, H_{\rm SF}$ remain approximately constant. Here the value for $H_{\rm FM}$ at 2 GPa was linearly extrapolated, from its values at 0 and 1 GPa, to be ~ 9.2 T. It is likely underestimated, since when we estimate it another way, by linearly extrapolating $R_{\rm xy}(p = 2\,\text{GPa})$ in Fig. 3(a) from the range $6 - 8.5$ T and look for the field where $-1\, e^2/h$ is likely reached, we get approximately 16 T.

Based on Eq. S9, $H_{\rm FM}$ increasing with pressure is only possible if H_E increases enough compared to the change in H_a, meaning

$$\delta H_E \cdot 4\cos^2(\pi/2N) > \delta H_a. \tag{S12}$$

In contrast, $H_{\rm c0}$ and $H_{\rm SF}$ attributed to the edges of the $\text{AFM}_{\pm 1}$ states for $H > 0$ are expected to increase monotonously with increasing H_a/H_a^c as shown in Fig. S12(d). Approximating either transition field linearly on the phase diagram, $H_{\rm c0(SF)}/H_{\rm FM0} \approx a + b \cdot H_a/H_a^c$ where $a, b > 0$. Consequently, $H_{\rm c0(SF)} \approx a \cdot H_{\rm FM0} + b \cdot H_a \cdot H_{\rm FM0}/H_a^c$ where the second term is positive and independent of H_E. $H_{\rm c0}, H_{\rm SF}$ being approximately independent of pressure requires that they do not change as H_E and H_a are varied by p. Based on the above, their

variations approximately relate as

$$\delta H_{\mathrm{c0(SF)}} \propto \delta H_E + c \cdot \delta H_a \tag{S13}$$

where $c > 0$. Therefore, the independence of pressure, i.e. $\delta H_{\mathrm{c0(SF)}} = 0$ requires that δH_E and δH_a have opposite sign. In conclusion, Eqs. S12, S13 lead to $\delta H_E > 0$ and $\delta H_a < 0$ as summarized in the main text.

Quantitative evaluation of H_E, H_a versus pressure:

The above appraisal is well matched by quantitative analysis. In Fig. S13(a), besides the experimental transition fields (solid markers), we also plot their fits (empty markers) based on the analytically predicted phase boundaries in Fig. S12(d). The model parameters H_E, H_a produced by the fits are plotted in Fig. S13(b). The former increases by approximately 25% with pressure, while the latter decreases by about 40%. We have also plotted the experimental fields in Fig. S13(c) (solid markers) over the theoretical phase diagram (solid lines), i.e. they have been rescaled by H_{FM0}. Pressure reduces the ratio H_a/H_a^c by half, moving all points lower on the graph. We emphasize that a larger value of $H_{\mathrm{FM}}(2\,\mathrm{GPa})$ produces an even more significant change in the parameters.

Since H_a/H_a^c is so low, based on the simulated colormap of Fig. S12(d) we expect a negligible hysteresis δ at the spin-flop (AFM-cAFM) transition, which is consistent with the Hall measurements in the main text.

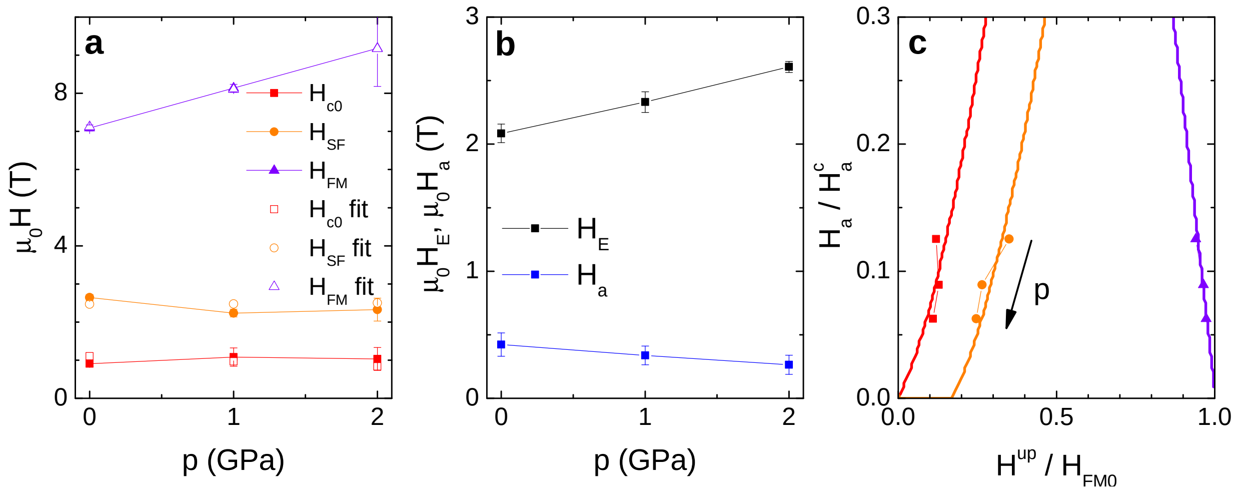

Figure S13: (a) The experimentally extracted transition fields vs pressure (solid symbols) and their fits (empty symbols). (b) The fit parameters H_E and H_a versus pressure. (c) The transition fields (solid symbols) rescaled by H_{FM0} on the analytical phase diagram. The solid lines are the same as in Fig. S12(d). The arrow indicates that H_a/H_a^c decreases with pressure.

References

(1) Hu, C.; Qian, T.; Ni, N. Recent progress in MnBi2nTe3n+1 intrinsic magnetic topological insulators: crystal growth, magnetism and chemical disorder. *National Science Review* **2024**, *11*, nwad282.

(2) Fülöp, B.; Márffy, A.; Tóvári, E.; Kedves, M.; Zihlmann, S.; Indolese, D.; Kovács-Krausz, Z.; Watanabe, K.; Taniguchi, T.; Schönenberger, C.; Kézsmárki, I.; Makk, P.; Csonka, S. New method of transport measurements on van der Waals heterostructures under pressure. *Journal of Applied Physics* **2021**, *130*.

(3) Lee, S. H. et al. Spin scattering and noncollinear spin structure-induced intrinsic anomalous Hall effect in antiferromagnetic topological insulator MnBi2Te4. *Physical Review Research* **2019**, *1*.

(4) Deng, Y.; Yu, Y.; Shi, M. Z.; Guo, Z.; Xu, Z.; Wang, J.; Chen, X. H.; Zhang, Y.

Quantum anomalous Hall effect in intrinsic magnetic topological insulator MnBi2Te4. *Science* **2020**, *367*, 895–900.

(5) Ovchinnikov, D. et al. Intertwined Topological and Magnetic Orders in Atomically Thin Chern Insulator MnBi2Te4. *Nano Letters* **2021**, *21*, 2544–2550.

(6) Yang, S.; Xu, X.; Zhu, Y.; Niu, R.; Xu, C.; Peng, Y.; Cheng, X.; Jia, X.; Huang, Y.; Xu, X.; Lu, J.; Ye, Y. Odd-Even Layer-Number Effect and Layer-Dependent Magnetic Phase Diagrams in MnBi2Te4. *Physical Review X* **2021**, *11*, 011003.

(7) Bac, S. K. et al. Topological response of the anomalous Hall effect in MnBi2Te4 due to magnetic canting. *npj Quantum Materials* **2022**, *7*, 1–7.

(8) Chen, B.; Liu, X.; Li, Y.-H.; Tay, H.; Taniguchi, T.; Watanabe, K.; Chan, M. H. W.; Yan, J.; Song, F.; Cheng, R.; Chang, C.-Z. Even-Odd Layer-Dependent Exchange Bias Effect in MnBi2Te4 Chern Insulator Devices. *Nano Letters* **2024**, *15*, 13.

(9) Wang, Z.; Gibertini, M.; Dumcenco, D.; Taniguchi, T.; Watanabe, K.; Giannini, E.; Morpurgo, A. F. Determining the phase diagram of atomically thin layered antiferromagnet CrCl3. *Nature Nanotechnology* **2019**, *14*, 1116–1122.
